# The five-year costs and benefits of extended psychological and psychiatric assessment versus standard intake interview for women with comorbid substance use disorders treated in compulsory care in Sweden

**DOI:** 10.1186/s12913-018-2854-y

**Published:** 2018-01-30

**Authors:** Tina M. Olsson, Mats Fridell

**Affiliations:** 1 School of Social Work, University of Gothenburg, Box 720, 405 30 Göteborg, Sweden; 2 Department of Psychology, Lund University, BOX 213, S-221 00 Lund, Sweden

**Keywords:** Substance dependence, Comorbidity, Incremental validity, Assessment utility, Clinical utility, Effectiveness, Economic evaluation

## Abstract

**Background:**

Women with comorbid substance use disorders are an extremely vulnerable group having an increased relative risk of negative outcomes such as incarceration, morbidity and mortality. In Sweden, women with comorbid substance use disorders may be placed in compulsory care for substance abuse treatment. Clinical intake assessment procedures are a distinct aspect of clinical practice and are a foundation upon which client motivation and continued treatment occurs.

**Method:**

The current study is a naturalistic quasi-experiment and aims to assess the five-year costs and benefits of a standard intake interview versus an extended psychological and psychiatric assessment for a group of chronic substance abusing women placed in compulsory care in Sweden between 1997 and 2000. Official register data on criminal activity, healthcare use, compulsory care stays and other services was retrieved and all resources used by study participants from date of index care episode was valued. In addition, the cost of providing the intake assessment was estimated.

**Results:**

Results show that the extended assessment resulted in higher net costs over five years of between 256,000 and 557,000 SEK per person for women placed in care via the Law on Compulsory Care for Substance Abusers (LVM). Higher assessment costs made up a portion of this cost. The majority of this cost (47–57%) falls on the local municipality (social welfare) and 11.6–13.7% falls on the individual patient.

**Conclusions:**

Solid evidence supporting the clinical utility or incremental validity of assessment for improving treatment outcomes in this setting was not confirmed.

## Background

Substance abuse and dependence is among the most prevalent, deadly and costly of health problems worldwide [1–4]. Clinical, epidemiological and general population studies repeatedly find that substance use disorders and psychiatric disorders co-occur in the general population and in treatment populations at higher than chance levels [5–8]. This clinical burden results in substantial individual suffering as measured for example by reductions in quality of life and disability adjusted life years [2, 9] as well as generating considerable costs for individuals and society [10–12].

Women with a comorbid substance abuse and psychiatric disorder have an increased relative risk of incarceration when compared with both general population women and men with comorbid substance abuse and psychiatric disorders [13, 14]. Research on comorbid substance use and psychiatric disorders among incarcerated populations shows that the majority of inmates worldwide suffer from a comorbid disorder [13, 15–17] and that the rate of mental health problems among prison populations is consistently higher than that found in the community [18–22] leading commentators to wonder if individuals with comorbid disorders are being given appropriate treatment or if they are instead being systematically criminalized [17]. In addition, comorbidity in substance dependent populations leads to increased risk for somatic and psychiatric health ailments, reduced social functioning and premature mortality [23–26]. In Sweden, women make-up approximately 25% of the substance abusing population and are over represented in treatment cohorts [27]. Understanding the clinical implications of routine services for substance abusing patients on important outcomes such as healthcare and criminal justice system involvement is paramount for future attempts to treat this vulnerable population.

Most individuals placed in compulsory care for substance abuse treatment in Sweden undergo a standard intake interview at admission (DOK, ADAD or ASI) and may undergo an extended psychological and psychiatric assessment (extended assessment). Intake and assessment procedures are distinct and unique aspects of clinical practice and consist of collecting various pieces of information on individual patients from various sources e.g., [28, 29]. Standard intake interviews and assessment procedures inform clinical practice and should be used to describe current functioning, select treatment goals, and provide feedback to individual clients c.f., [29–32]. Assessment of individuals placed in compulsory care in Sweden is intended to be the foundation upon which client motivation and continued treatment occurs. It is intended to provide a well-documented and thoroughly thought through plan for treatment [33]. Another important aim of assessment is to avoid referring clients to treatment interventions which could be unsuitable or harmful. Implicit in the decision to collect data from multiple sources is the notion that doing so translates into improved client outcomes that may outweigh the added cost of the additional data collection or add information which could guide further clinical activities [34, 35]. Over the past 15 years there has been an increased interest in collecting background data and carrying out diagnostic assessment as well as understanding if these would be gains outweigh the additional costs associated with clinical assessment.

The clinical utility [36, 37] of assessment has been repeatedly called into question. While there is in some cases strong empirical evidence supporting the predictive validity of standardized interviewing procedures e.g., psychological testing; [29], the clinical utility as well as the incremental validity (added benefit) of these procedures is all but unknown [28, 30, 34, 38, 39]. The purpose of this study, therefore, is to assess the incremental validity of a standard intake interview versus an extended psychological and psychiatric assessment by empirically investigating the costs and benefits generated over a five-year follow-up period for a group of chronic substance abusing women placed in compulsory care in Sweden between 1997 and 2000.

Healthcare costs, crime costs, and productivity losses are consistently identified in international research as the largest contributors to the economic costs of substance abuse [10, 11, 40, 41]. Distinct aspects of intervention and treatment, such as assessment, within the real world of substance abuse treatment are ultimately aimed at reducing substance use, improving levels of overall functioning and health, and or improving public safety and crime outcomes [42, 43]. These methods have the potential of not only improving the lives of individuals but of providing benefit to society through a reduction in healthcare costs, crime costs and productivity losses. This potential, however, is not a given and care must be taken in empirically assessing the extent to which policy impacts individual, population, and system health.

The current study is the fourth and final study in a series of economic studies of this group. The first study investigated the long-term pattern of criminal justice system involvement and costs incurred over a 30-year period for this group [44]. The second study investigated productivity losses associated with criminal behavior for this group [45]. The third study described the group’s use of direct healthcare resources and related productivity losses over a 32-year period [46]. The current study aims to answer the following question:

- What are the five-year incremental costs and benefits of extended assessment compared to standard intake interviews for chronic substance abusing patients?

## Method

### Design

The economic evaluation is based on a quasi-experiment with natural allocation between a group receiving a standard intake interview (standard practice) and a group receiving the extended psychological and psychiatric assessment. Assessment of baseline characteristics between the groups receiving the standard intake interview and extended assessment at intake was completed on a range of variables. Full details of these analyses are reported elsewhere [47, 48]. Briefly, psychosocial variables, treatment history variables, diagnostic variables, substance abuse variables, criminal involvement, as well as family characteristics and history were compared and assessed. As original background data was not available for the current study, significant differences in the above variables are re-reported. Importantly, no differences were found in those analyses in groups at baseline on the outcomes assessed here. The long-term outcomes reported in this study are based entirely on official register data. In the current study, the standard t-test is used to assess differences between groups on all resources used over the 5-year follow-up period. Statistical differences are then valued monetarily. Differences in costs or benefits between groups are assessed using the standard t-test. Absent statistical differences on resource use in the measured outcome areas, subsequent benefits are valued at zero.

### Setting

#### Compulsory Care in Sweden

In Sweden, youth with serious psychosocial problems and adult substance abusers can be remanded to compulsory care. Individuals are admitted to compulsory care under the Law on Compulsory Care for Substance Abusers (LVM, act 1988:870) or The Care of Young Persons Act (LVU, act 1990:52). According to the LVU, “A care order is to be issued, if the young person exposes his health or development to a palpable risk of injury through the abuse of addictive substances, criminal activities, or some other socially degrading behavior.” (LVU, act 1990:52, section 3). Youth can also be taken into care under the LVU due to neglect or chaotic circumstances in the family. Under Section 4 of the LVM, a court can order compulsory care for a person whose health is deemed to be at risk, or who may be placing others at risk, and who is considered to need assistance in order to discontinue substance use. The LVM and LVU acts are unrelated to penal code or laws of psychiatric care. Social welfare, or, more rarely, police, family members or a general practitioner can report patients to the court. Within 8 days after report, an assessment of need for treatment or physician evaluation must be completed, and court hearings proceed. Care orders are implemented in specially certified institutions, under the authority of the National Board for Institutional Care (SiS). The clinical services that are provided to individuals placed in compulsory care are not specified by the LVU or the LVM but are based on clinical decisions made within the institutional environment following intake and assessment. There are currently 36 SiS institutions nationwide. Upon entry into compulsory care, all individuals undergo a standard intake interview. The local social welfare administration, however, can request SiS to conduct an extended assessment in addition to the standard intake interview. The number of women undergoing compulsory care annually in Sweden is approximately 650–700 [49].

#### Lunden

Lunden is a 21-bed SiS-run inpatient compulsory care residential treatment unit. The institution has 12 beds for adult women and 9 beds for female youth under 20 years of age. Lunden has a national catchment area. At the time of this study, the treatment unit was reserved for treatment of women exclusively, with a focus on women’s issues and drug addiction. The unit employs a range of clinical and administrative staff [50–52].

### Subjects

All patients admitted to Lunden from January 1, 1997 to December 31, 2000 (*n* = 227) were consecutively included in the cohort followed in this study. Participants were followed from intake to 5-years post intake. At intake, 92 of the women (40.5%), were remanded to compulsory care via the LVU and 135 (59.5%) to compulsory care via the LVM. All participants completed the standard intake interview and a subsample of participants (*n* = 130) underwent an extended psychological and psychiatric assessment in addition to the standard intake interview. Although women were remanded to care and subsequently entered into the study due to their substance abuse, it was found after entry into the study that a total of 78% of the women had at least one personality disorder according to SCID-II DSM-IV; [53] interviews, and 42% had an axis I disorder in addition to the substance use diagnoses. The diagnostic spectrum of psychiatric problems in this cohort is one of major comorbid diagnosis with personality disorder (78%), anxiety- and depressive disorders (36%), and, to a lesser degree, psychoses (Schizophrenia 5% and substance related psychosis 15%) [8, 47, 48]. The main drug used by participants prior to admission was stimulants, mainly amphetamine (51%), opiates, mainly heroin (35%), alcohol (7%), or other drugs (all < 3%). This pattern of poly-substance use is common for clinical samples of drug dependent populations in Sweden. Unique to this sample was that 93% used primarily narcotic drugs while a diagnosis of alcohol dependence was less common (9%) [47]. The cohort followed in this study can be characterized as one with severe and long lasting psychiatric disorders, social problems and a chronic substance abuse with a relatively small number of patients with psychosis. The mean age of the subjects at intake to treatment was 18.7 years for the LVU subjects (range: 16–20) and 26.7 for the LVM subjects (range: 18–43) [47, 48]. Of the women followed in this study, 92.9% have been charged for at least one crime [44, 48, 51] and 22 (9.7%) died during the 5-year follow-up period, prior to January 1, 2007 (4 LVU; 18 LVM) (4.3%LVU, 13.3% LVM) at the average age of 34.5 years. In the cases of premature death, cause of death as reported in forensic autopsy reports was substance use in 18 cases and somatic disease in four cases.

### Procedure

All individuals remanded to compulsory care are assigned to a facility through a central placement office in Stockholm, Sweden. Individuals are placed in institutions nationwide based on availability and services offered. All women placed at Lunden during the period under review were consecutively included in the study. At intake, all women go through detoxification/abstinence treatment and then complete the standard intake interview. A sample of women received the extended psychological assessment as per request from their local municipal case worker. SiS, Lunden, and/or research staff had no influence on whether an individual was placed at Lunden or whether they subsequently received a standard intake interview alone or an extended psychological assessment.

### Cost: Standard intake interview

Information on the time demands of all SiS staff for completion of the standard intake interview was collected directly from SiS. Time was valued using Statistics Sweden (2014) salary statistics across Sweden by title.

#### LVU

For the women mandated to compulsory care via the LVU, the standard intake interview is based on the Adolescent Drug Abuse Diagnosis ADAD, [54]. This questionnaire is used in all LVU cases in SiS institutions nationwide. The ADAD covers nine substantive areas: somatic health, education, work and leisure time, friends, family, mental health, criminal behavior, alcohol and drug use. The ADAD is carried out through interview. All interviewers are trained in the use and properties of the instrument. The instrument has been validated on Swedish samples [55].

#### LVM

All women mandated to compulsory care via the LVM complete the Swedish developed evaluation and documentation system [DOK, [56]; c.f. Addiction Severity Index ASI]. The instrument is implemented in all LVM cases in SiS institutions nationwide. DOK covers nine substantive areas: background, work, alcohol and drug use, treatment history, somatic health, mental health, criminal activity, agency and other treatment contacts, and help and support.

### Cost: Extended psychological and psychiatric assessment

A subsample of 130 women (52 LVU, 78 LVM) (57%; 56% LVU; 57% LVM) underwent the extended psychiatric and psychological assessment upon referral to SiS. Information on the time demands for completion of the extended assessment was collected directly from SiS. Time was valued using Statistics Sweden (2014) salary statistics across Sweden by title.

#### LVU extended psychological and psychiatric assessment

For LVU placed adolescents, the extended psychological assessment was carried out by combining data from interviews, professional assessment and psychological testing, and continuous observation during an eight to ten-week period. Assessment teams carry out LVU extended assessments. The assessment coordinator had a coordinating role and the psychologist conducted interviews and tests in collaboration with a part-time visiting psychiatrist. The assessments were carried out in close collaboration with the referring social welfare agency. The assessment’s starting point was the standard intake interview complemented by clearly defined assessment questions with clear application to service need, education, and the immediate situation regarding social and psychological problems. Ongoing feedback on the assessment process and assessment results was reported by the assessment team to the adolescent, his or her parent/guardian(s) as well as to the referring social welfare agency.

In addition to the areas covered in the standard intake interview, the extended assessment includes information collected on behavioral observations in the institutional environment, interviews with family members and other members from the adolescent’s network, psychological assessment, neuropsychological assessment, autism spectrum assessment, and other common child and adolescent diagnostics. Evaluation is made on the individual’s general aptitude; personality disorders; criminal behavior; ADHD, autism, and other diagnoses; anxiety, anger, conduct disorders, depression and self-esteem; evaluation of statements from family and other close contacts and childhood experiences.

The following standardized tests and psychiatric assessment instruments were used when there was clinical indication of a given problem. All tests have been validated on Swedish groups and were selected according to the norms and standards relevant to each age group. For all tests, Swedish translations were used. Although the most recent version of the instrument is referenced below, earlier versions may have been in used at the time of this study:

1. Wechsler Intelligence Scale for Children, 4th edition (WISC-IV). Intelligence test for children aged 6–16; or the Weschsler Adult Intelligence Scale, 4th edition (WAIS-IV). Intelligence test for individuals aged 16–90; [57, 58].
2. Delis Kaplan Executive Function System D-KEFS, [59]. Assesses executive function in individuals aged 8–89 years. In addition, attention problems in individuals aged 6+ years were assessed using Conners’ Continuous Performance Test II, version 5 CPT II V.5, [60]. Test of Variables of Attention (TOVA) was used as an alternative to CPT II [61].
3. Wisconsin Card Sorting Test (WCST). Assesses perseveration and abstract reasoning [62].
4. Rey Complex Figure Test and Recognition Trial (RCFT). Assess visuospatial ability and visuospatial memory in individuals aged 6–89 years [63].

#### LVM extended psychological and psychiatric assessment

For LVM placed women, the extended assessment was carried out by way of information collection, clinical interviews, psychological and psychiatric assessments and tests, psychological tests and continuous observation in the institutional environment over an eight to ten-week period. Assessment teams may consist of a social worker, physician or nurse, psychologist, or part-time visiting psychiatrist. The psychologist or psychiatrist conducted the clinical interviews and testing in collaboration with the assessment team. The assessment findings were shared with the referring social welfare agency and the patient being assessed received ongoing feedback regarding progress and results.

In addition to the areas covered in the standard intake interview, the extended assessment included behavioral observations in the institutional environment, psychological assessment, and neuropsychological assessment. Depending on the presenting problems of the patient, the structured test battery included the WAIS-IV [58], D-KEFS, CPT II V.5 [59] or alternatively TOVA [61], WCST [62], and the RCFT [63]. In addition, the Wechsler Memory Scales WMS III, [58] were used to measure adult memory, the Rey Auditory Verbal Learning Test RAVALT; [64–66] or the Claeson-Dahls intelligence and memory test [67] was used to measure immediate recall, and the Structured Clinical Interview for DSM IV, axis II SCID II; [68] was used to diagnose axis II personality disorders. The LVM assessment and its impact on treatment planning has been investigated previously [32].

### Benefits: Behavioral effects

#### Crime outcomes

The estimation of longitudinal criminal justice system (CJS) resource use and costs for this group of women has been detailed elsewhere [44, 45]. Briefly, official register data was collected which included information on criminal charges, prosecutions, sentencing and institutional care following criminal behavior for the period under review. The number of crimes committed per participant during the period under review was recorded and tested for differences between groups. Resource use could be estimated for eight types of crimes: violent crimes including robbery; other crimes toward the person; theft and larceny; narcotics offenses; traffic offenses; vandalism and other property crimes; fraud; and other crimes. Resource use impacting six actors in the CJS were included: police, prosecutors, court system, prison and probation services, psychiatric care, and state institutions for juvenile detention and care of adult substance abusers. In addition, productivity loss as a result of incarceration and homicide as well as tangible and intangible victim costs were included in crime cost estimates.

#### Healthcare outcomes

The estimation of longitudinal hospital care resource use and costs for this group has been detailed elsewhere [46]. Briefly, official register data was collected which included information on hospital inpatient and outpatient care by primary diagnosis (ICD, international classification of diseases) for the period under review. The cost per patient per primary diagnosis (ICD-10) was valued using national data from Sweden’s cost per patient database (KPP). In addition, productivity losses due to inpatient stays and lost life were included.

#### Compulsory care outcomes

Data regarding women placed in compulsory care under the LVM were retrieved reporting the dates of index care (study intake), placement and discharge. According to the Swedish National Board for Institutional Care [33], 70% of the cost of placing individuals via the LVM was financed by charging the municipal social services directly. In 2010, the average daily cost for placement via the LVM was 5025 SEK. For the LVU group, data was retrieved regarding the date of index care. No information was available, however, regarding the discharge date for patients receiving LVU services. According to SiS the average length of stay (LOS) for girls in compulsory care and placed via the LVU was 129 days and the daily cost for placement of girls who received compulsory care via the LVU was 7407 SEK during 2010, 67% of which was financed by charging the municipal social services directly (ibid.).

#### Paragraph-27 care

For women placed via the LVM, the goal of treatment is to transition individuals as quickly as possible from institutional care to more open forms of treatment outside of the state institution. This is referred to as paragraph-27 care. Paragraph-27 care can take on many forms and can be offered as a placement intervention such as placement at a group home or foster home or may be offered as a non-placement intervention such as counseling support for example. These interventions are provided by the municipal social services with support (oversight) from SiS. Information on the costs of services provided within the municipal social services is difficult to obtain, as this data is not routinely tracked as part of on-going municipal record keeping [69–71]. Therefore, estimates of the unit cost of providing various forms of prevention and intervention activities within the municipal social services in Sweden are taken from two prior studies [69, 70] and applied to resource use data collected on the LVM group followed in this study. The average unit cost applied for paragraph-27 care was as follows: group home placement 3322 SEK, foster home placement 1373 SEK, non-placement services 665 SEK, SiS oversight 625 SEK.

#### Productivity

There are several methods available for estimating productivity loss [72, 73]. Changes in productivity in this study are valued using the human capital approach [74] and as such follows the methodology used in prior studies of this group [44–46]. This approach values changes in productivity as the present value of lost (or freed) time according to the market wage. The potential impact on individual productivity as estimated by changes in compulsory care stays is valued using the average monthly wage for women across Sweden by age in 2010 [75]. Changes in productivity are presented separately in the cost-benefit analysis and may, therefore, be removed or amended by the interested reader.

### Cost-benefit analysis

Differences in costs and benefits of extended psychological and psychiatric assessment are tested using the standard *t*-test. Statistically significant differences are aggregated by way of the basic economic equation for calculating net present value (NPV) as follows:

$$ {NPV}_{intake}=\sum \limits_{t= intake}^N\frac{B_t}{{\left(1+i\right)}^t}-\sum \frac{C_t}{{\left(1-i\right)}^t} $$

In this model, NPV is equal to the value of the significant effects per participant (*B*) during a given year (*t*) minus the cost for providing the extended psychological and psychiatric assessment per participant (*C*) during a given year. These values are measured from the day that the individual entered the study and was assigned to an assessment group and five years forward (*N*). Future costs and benefits are discounted back to the day that an individual entered the study with help of a discount rate (*i*). Due to the theoretical variation in choice of discount rate, all results are presented without discounting as well as with a 3.5% discount rate as recommended by Moore, Boardman, Vining, Weimer, & Greenberg (2004) [76]. In this analysis, results are presented from three perspectives: participants, others and society.

### Currency, inflation, and time effects

Cost estimates were calculated in Swedish crowns (SEK) and reported to the nearest 1000 SEK (100 SEK for values under 1000 SEK). For comparison purposes, the purchasing power parity (PPP) based exchange rate in 2010 was 9.03 SEK = 1.00 US Dollar = 0.76 Euro [77]. Unit costs are estimated for year 2010 and applied to resource use at the individual level. Unit costs for paragraph-27 care were inflated from estimated values [69, 70] using the change in service price index (TPI: 2005 = 100; 2006 = 101; 2007 = 103; 2010 = 109; Statistics Sweden, 2013).

### Sensitivity analysis

The benefit estimate as reported in this study was further assessed using the non-parametric bootstrap [78]. Here, the sample was first stratified and then a random sample was drawn 1000 times from the range of individual benefit estimates to arrive at an empirical estimate of total benefit.

## Results

### Differences in baseline characteristics

Few differences were found between the groups of women that received the standard intake interview compared to the extended psychological assessment at intake. In the LVM group, the women that received an extended psychological assessment were somewhat younger (M, 26.8 years; SD, 5.8 years) than those women who received the standard intake interview only (M, 29.5 years; SD, 8.5 years; *p* ≤ .05). No other differences were found between women placed via the LVM. In the LVU group, the women that received an extended psychological assessment had committed on average more crimes prior to intake (M, 4.7; SD, 3.1) than the group that received the standard intake interview (M, 2.2; SD, 2.4; *p* ≤ .01). In addition, women in the LVU group that received the extended psychological assessment were less likely to come from substance abusing families (43 versus 53, *p* ≤ .05) but more likely to have had a family member with a psychiatric disorder during childhood (30 versus 20, *p* ≤ .05), more likely to live alone (20 versus 18, *p* ≤ .05), and used opiates to a greater extent (27 versus 15, *p* ≤ .05) than those women who received the standard intake interview alone. There were no differences between groups in drug use or length of drug use, age of debut or other social problems.

### Incremental assessment costs

Table 1 summarizes the professional, participant, and other actors’ time demands and total costs for completing the standard intake assessment versus the extended psychological assessment. Total incremental assessment costs for the LVU extended psychological assessment was estimated to be 45,000 SEK per person and total incremental assessment costs for the LVM extended psychological assessment was estimated to be 55,000 SEK per person.

**Table 1:** Standard intake psychological testing versus extended psychological assessment time and cost (SEK, 2010)

	Monthly salary, SCB (hourly equivalent, including social fees) (a)	Time (hours)				Incremental cost extended psychological assessment^a^	
		LVU		LVM		LVU (f) = [(c)-(b)]*a	LVM (g) = [(e)-(d)]*(a)
		Standard Intake Testing (b)	Extended Psychological Assessment (c)	Standard Intake Testing (d)	Extended Psychological Assessment (e)		
Case worker	26,800 (203)	3	114	5	103.5	23,000	20,000
Psychologist	34,500 (261)	0	35.5	0	56	9000	15,000
Social worker	27,500 (208)	0	12.5	0	9	3000	2000
Physician	46,300 (350)	0	5	0	9	2000	3000
Nurse	35,200 (266)	0	5	0	26.5	1000	7000
Participant	20,600 (156)	3	34	5	54.5	5000	8000
Parent/guardian/other family	28,400 (215)	0	13.5	0	5	3000	1000
Total^b^	–	6	219.5	10	263.5	45,000	55,000

^a^All amounts rounded to nearest 1000SEK

^b^May not be equal to sum of column due to rounding

### Compulsory care costs

The average length of (index) stay in compulsory care for women mandated to care via the LVM was 85 days (SD 54). Average total costs per participant for the index stay in compulsory care was 439,000 SEK (SD 274,000 SEK). The LVM participants that received and extended assessment stayed significantly longer in compulsory care (106 days, SD 45 days) compared to the participants that received the standard intake interview only (57 days, SD 54 days; *p* ≤ 0.01) (Table 2). The difference in compulsory care costs between LVM groups was 157,000–333,000 SEK per person (95% CI, Table 3).

**Table 2:** Changes in Resource Use following Extended Psychiatric Assessment versus Standard Intake, LVU, LVM and Lunden Groups (SEK, 2010)

	LVU (*n* = 92)					LVM (*n* = 135)					Lunden (*n* = 227)				
	Extended Psychological Assessment (*n* = 52)	Standard Intake (*n* = 40)	*t*	*p*	95% CI (diff.)	Extended Psychological Assessment (*n* = 78)	Standard Intake (*n* = 57)	*t*	*p*	95% CI (diff.)	Extended Psychological Assessment (*n* = 130)	Standard Intake (*n* = 97)	*t*	*p*	95% CI (diff.)
Compulsory Care															
Length of Stay – Lunden^a,b^	–	–	–	–	–	106 (45)	57 (54)	5.536	.00	31–66	–	–	–	–	–
Paragraph 27 care															
Days in p27 care^a,b^	–	–	–	–	–	55 (43)	29 (46)	3.318	.00	10–42	–	–	–	–	–
Crime															
Total Arrests	3.15 (2.86)	2.83 (3.15)	.524	.60	−.918–1.58	3.37 (3.79)	3.68 (3.82)	−.472	.64	−1.62 - .99	3.28 (3.44)	3.33 (3.57)	−.097	.92	−.97–.88
Total Crimes committed	7.30 (9.53)	7.90 (9.90)	−2.9	.77	−4.64 – 3.46	7.76 (10.65)	10.96 (18.06)	−1.28	.20	−8.10 – 1.71	7.58 (10.19)	9.70 (15.24)	−1.25	.21	−5.44 – 1.21
Violent crimes and crimes against the person	.50 (.82)	.37 (.66)	.77	.43	−.19–.44	.24 (.68)	.47 (.90)	−1.60	.11	−.51–.05	.34 (.75)	.43 (.81)	−.82	.40	−.29–.11
Non-violent crimes	2.65 (2.44)	2.45 (3.07)	.35	.72	−.93–1.34	3.12 (3.65)	3.21 (3.80)	−.12	.89	−1.36 – 1.19	2.93 (3.21)	2.89 (3.52)	.09	.92	−.84–.92
Healthcare															
Days in hospital – total	35 (110)	26 (66)	−.44	.66	−47 - 30	50 (175)	35 (69)	−.59	.55	−63 - 34	44 (152)	32 (67)	−.74	.46	−45 – 20
Days in hospital – somatic care	15 (58)	8 (12)	−.76	.45	−26 - 11	16 (33)	12 (23)	−.84	.40	−14 - 6	16 (44)	10 (19)	−1.12	.26	−15 - 4
Days in hospital – psychiatric care	20 (91)	19 (64)	−.10	.92	−35 - 32	34 (174)	23 (64)	−.43	.67	−58 - 38	28 (146)	21 (64)	−.44	.66	−38 -24

^a^Length of stay in compulsory care and paragraph-27 care available for LVM women only

^b^All results rounded to the nearest day

**Table 3:** Changes in Direct Costs and Productivity following Extended Psychiatric Assessment, LVM Group and Sensitivity Analysis^a^ (SEK, 2010)

	LVM (*n* = 135)				
	Extended Psychological Assessment (*n* = 78)	Standard Intake (*n* = 57)	*t*	*p*	95% CI (diff.)
Changes in Direct Costs and Productivity					
Total Cost-Lunden	533,000 (225000)	288,000 (273000)	5.536	.00	157,000–333,000
Total Cost – p27 care	148,000 (155000)	94,000 (168000)	1.911	.06	−3000–109,000
Productivity Loss	91,000 (39000)	49,000 (47000)	5.536	.00	27,000–57,000
Sensitivity Analysis	95% CI mean estimate	95% CI mean estimate	Mean diff.	*p*	95% CI (diff.)
Total Cost - Lunden	483,000–583,000	220,000–363,000	245,000	.00	155,000–330,000
Total Cost – p27 care	113,000–183,000	54,000–137,000	53,000	.06	−400 – 109,000
Productivity Loss	83,000–100,000	38,000–62,000	42,000	.00	27,000–57,000

^a^Results rounded to nearest 1000 SEK (100 SEK for values less than 1000 SEK)

### Paragraph-27 costs

The average number of days in paragraph-27 care during index care episode was 44 days (SD 46 days) per person in the LVM group. Total costs for paragraph-27 care initiated during index care episode were 125,000 SEK (SD 162000 SEK). Participants that received an extended assessment stayed significantly longer in paragraph-27 care compared to those participants that received the standard intake interview only (Table 2). Differences in cost between the two groups for paragraph-27 care was − 3000–109,000 SEK per person (95% CI) with the group receiving the extended assessment incurring more costs than the comparison group (Table 3).

### Crime costs

Participants were arrested, on average, 3.30 times (SD 3.48; LVU 3.01, SD 2.97; LVM 3.50, SD 3.79) during the five-year follow-up period. Participants committed on average 8.48 (SD 12.61; LVU 7.56, SD 9.65; LVM 9.11, SD 14.28) crimes during the period under review. There were no differences found in the number of arrests or number of crimes committed between the group of women that received the extended assessment versus those that received the standard intake interview only. In addition, there were no differences found between groups in the extent to which violent crimes and crimes against the person or non-violent crimes were committed (Table 2).

### Healthcare costs

Participants spent on average 38.69 days (SD 123.06; LVU 31.63, SD 67.43; LVM 43.96; SD 151.93) in hospital during the five-year follow-up period. Of these total days, 25.38 days (SD 118.02; LVU 21.43, SD 64.06; LVM 28.33, SD 146.04) were spent in psychiatric care and 13.31 days (SD 35.92; LVU 10.20, SD 19.10; LVM 15.63, SD 44.46) were spent in somatic care. There were no differences in the number of days spent in hospital between the two groups (Table 2).

### Productivity loss

Average productivity losses for individuals placed in compulsory care via the LVM were 73,700 SEK (SD 47000) per person. Due to the significant increase of days in compulsory care for the subsample placed via the LVM for the group of participants who completed an extended assessment versus a standard intake interview, the difference in productivity loss between groups was valued between 27,000 and 57,000 SEK per participant placed via LVM (Table 3).

### Sensitivity analysis

Results of the sensitivity analysis are presented in Table 3. There was little change in the 95% CI around the difference in benefit between the main analysis and the sensitivity analysis.

### Cost-benefit analysis

The group of women who received an extended assessment had a higher net cost to society over five years of between 256,000 and 557,000 SEK per person placed via LVM. The majority of this cost (47–57%) falls on the local municipality (social welfare) and 11.6–13.7% falls on the individual patient.

## Discussion

The purpose of this study was to assess the five-year costs and benefits of extended psychological and psychiatric assessment versus standard intake interview for a group of chronic substance abusing women placed in compulsory care for substance abuse between 1997 and 2000 and is the first study of its kind within the compulsory care system in Sweden. Results showed that after five-years there were no differences between groups in criminal involvement or healthcare use. Those women that received an extended assessment had on average longer stays in both compulsory care and paragraph-27 care, which translated to increased direct costs and productivity losses when compared to the women that underwent a standard intake interview only. This combined with the increased incremental cost of the extended psychological and psychiatric assessment led to an additional cost of 256,000–557,000 SEK per person (LVM only).

Solid evidence supporting the clinical utility or incremental validity of assessment for improving treatment outcomes is lacking [28, 37, 79–82]. Still, an enormous amount of resources are being used for data collection efforts for assessment purposes —resources that could be used for treatment and/or other clinical activities. Therefore, careful consideration must be made in assessing when additional assessment activities provide benefit. In the group studied here, assessment was made at the request of the referring social worker, due to observed indication of severe psychological or mental problems, with the goal of influencing treatment planning. In this study, assessment activities required approximately 26 times the hours necessary for the standard intake interview alone with an absolute difference of over 200 man hours (Table 1). This is substantially greater than the amount of time afforded to clients for many psychosocial treatment services when they are offered. Psychosocial treatments that have some evidence of effectiveness among substance using individuals [83–88] or have promise for the effective treatment of clients with a co-occurring disorder [89] and that fall under national guidelines for the treatment of substance abuse [90] are largely interventions which are provided between 5 and 20 sessions. Often, these interventions have not been found effective for severe substance abusers such as those followed in this study [88]. Most of the patients in compulsory care have problem levels where 5–20 sessions are seldom realistic and their compliance and retention in outpatient care is very low [91]. Further, in a national study of psychosocial treatment methods in Sweden, it was found that few patients (5–50%) receive any structured interventions [92]. In this sample of women, only about 10% had ever been offered or started a psychotherapeutic treatment or completed a pharmacological intervention. Importantly, evidence based pharmacological or psychosocial treatments are not available for amphetamine users which was the major problem in this particular sample [88]. However, the longer stays in compulsory care and paragraph-27 care by the group receiving the extended psychological and psychiatric assessment may be indication of better retention or increased motivation of this group. Unfortunately, we were unable to assess the extent to which patients followed treatment planning and whether this had bearing on outcome.

Understanding how clinical practice impacts client outcomes is an important aspect of public health policy, practice and research. Studies that attempt to assess the impact of “real world” clinical practice must also attempt to place results thoughtfully in the context of real world practice. Although this study has found that the incremental effectiveness of extended psychological and psychiatric assessment as examined in this study does not provide strong justification for the additional cost necessary to undertake the extended assessment, one must bear in mind that assessment as used in clinical practice in Sweden is not intended to be therapeutic but instead is intended to drive intervention and treatment decisions. Clinical decision-making is a complex task. It is not only impacted by practitioner characteristics, but is also subject to a string of biases [93–95] and has been under scrutiny for at least 60 years [96, 97]. The results of this study indicate that there may be shortcomings in the extent to which social workers and mental health clinicians are able to translate the findings from extended assessments into real life treatment planning. These findings may also be indicative of the need for more high quality interventions for this group of clients as extended psychological and psychiatric assessment, currently, does not automatically yield better results for this group of clients. As this study did not assess the extent to which clinicians use interview and assessment data once collected we are unable to assess whether the information collected is being used in case planning.

As the ultimate identification, selection, and provision of therapeutic services to this vulnerable group rests on the quality of the case formulation and diagnostic procedures upon which treatment decisions are based it is crucial to ensure that the interview and assessment procedures that clinicians use in routine practice are of high-quality and have demonstrated clinical utility. Still, the investigation here should not be interpreted as having bearing on the clinical efficacy of extended assessment. This study did not control for the types of interventions and/or treatments participants received subsequent to initial interview and assessment and therefor speaks only to the effectiveness of extended assessment as compared to the standard intake interview as currently used in routine practice. Any attempts to assess the efficacy of extended assessment would need to control for the types of services and interventions participants receive post-assessment. In Sweden and internationally, however, little is known about the extent to which psychosocial interventions are effective in improving important clinical outcomes for chronic substance abusing women with co-occurring disorders. Ultimately, improvements in assessment methods should lead to better therapy and aftercare. The results of this study indicate, however, that extended assessment may not automatically lead to immediate clinical gains.

It should be stressed that this study was a quasi-experiment with natural allocation between comparison groups and therefore placed no controls on which participants received the standard intake interview or the extended psychological assessment. Additionally, as original clinical data was unavailable for the economic evaluation, moderator analysis was not performed on those variables found to differ between the experimental groups compared in this study. As such it is unknown as to whether these differences had a moderating effect on outcome.

## Conclusions

Solid evidence supporting the clinical utility or incremental validity of extended psychological and psychiatric assessment for improving treatment outcomes for women with comorbid substance use disorders in this setting was not confirmed in this study. In addition, concerns are raised regarding the extent to which current practice benefits this vulnerable group. Further research should be aimed at gaining a deeper understanding of the clinical efficacy of assessment practices, how assessment information is translated and applied in clinical practice, and the clinical effectiveness of the treatment options provided this group following assessment.

## Abbreviations

ADAD: Adolescent Drug Abuse Diagnosis
ADHD: Attention Deficit Hyperactivity Disorder
ASI: Addiction Severity Index
CJS: Criminal Justice System
CPT II: Conners’ Continuous Performance Test II, v. 5
D-KEFS: Delis Kaplan Executive Function System
DOK: Documentation system within substance abuse treatment
DSM-IV: Diagnostic and Statistical Manual of Mental Disorders v. IV
ICD: International Classification of Diseases
KPP: Cost per Patient Database
LOS: Length of Stay
LVM: Law on Compulsory Care for Substance Abusers
LVU: The Care of Young Persons Act
NPV: Net Present Value
PPP: Purchasing Power Parity
RAVALT: Rey Auditory Verbal Learning Test
RCFT: Rey Complex Figure Test and Recognition Trial
SCID-II: Structured Clinical Interview for DSM disorders v. Ii
SEK: Swedish Crowns
SiS: National Board for Institutional Care
TOVA: Test of Variables of Attention
TPI: Service Price Index
WAIS-IV: Wechsler Adult Intelligence Scale, 4th edition
WCST: Wisconsin Card Sorting Test
WISC-IV: Wechsler Intelligence Scale for Children, 4th edition
WMS III: Wechsler Memory Scales
